# Present Molecular Limitations of ON-Bipolar Cell Targeted Gene Therapy

**DOI:** 10.3389/fnins.2017.00161

**Published:** 2017-03-29

**Authors:** Michiel van Wyk, Elmar C. Hulliger, Lara Girod, Andreas Ebneter, Sonja Kleinlogel

**Affiliations:** ^1^Institute of Physiology, University of BernBern, Switzerland; ^2^Department of Ophthalmology, Inselspital, Bern University Hospital, University of BernBern, Switzerland

**Keywords:** optogenetic vision recovery, bipolar cells, gene therapy, AAV vectors, human retina, rd1 mouse model, rd10 mouse model, expression pattern analysis

## Abstract

Recent studies have demonstrated the safety and efficacy of ocular gene therapy based on adeno-associated viral vectors (AAVs). Accordingly, a surge in promising new gene therapies is entering clinical trials, including the first optogenetic therapy for vision restoration. To date, optogenetic therapies for vision restoration target either the retinal ganglion cells (GCs) or presynaptic ON-bipolar cells (OBCs). Initiating light responses at the level of the OBCs has significant advantages over optogenetic activation of GCs. For example, important neural circuitries in the inner retina, which shape the receptive fields of GCs, remain intact when activating the OBCs. Current drawbacks of AAV-mediated gene therapies targeting OBCs include (1) a low transduction efficiency, (2) off-target expression in unwanted cell populations, and (3) a poor performance in human tissue compared to the murine retina. Here, we examined side-by-side the performance of three state-of-the art AAV capsid variants, AAV7m8, AAVBP2, and AAV7m8(Y444F) in combination with the 4x*GRM6*-SV40 promoter construct in the healthy and degenerated mouse retina and in human *post-mortem* retinal explants. We find that (1) the 4x*GRM6*-SV40 promoter is *not* OBC specific, (2) that all AAV variants possess broad cellular transduction patterns, with differences between the transduction patterns of capsid variants AAVBP2 and AAV7m8 and, most importantly, (3) that all vectors target OBCs in healthy tissue but *not* in the degenerated *rd1* mouse model, potentially limiting the possibilities for an OBC-targeted optogenetic therapy for vision restoration in the blind.

## Introduction

Blindness caused by photoreceptor degeneration affects approximately 1 in 3000 people world-wide. Since the inner retinal layers typically remain intact after photoreceptor loss, novel optogenetic therapies have aimed to recover light sensitivity in the inner retina through exogenous expression of light sensitive ion channels. When introduced to the retinal ganglion cells (GCs) or ON-bipolar cells (OBCs) of blind *rd1* mice, Channelrhodopsin-2 (ChR2) restored light sensitivity and basic levels of visual processing (Bi et al., 2006; Lagali et al., 2008; Doroudchi et al., 2011).

The first Phase I\IIa clinical trial on human patients (NCT02556736), which targets at delivering ChR2 to GCs, was launched in 2016. In parallel, the unnaturally high light intensities required for ChR2 activation have spurred the development of novel optogenetic tools based on more light-sensitive mammalian proteins (Cehajic-Kapetanovic et al., 2015; van Wyk et al., 2015a). These next-generation optogenetic therapies target the OBCs and re-activate the native OBC signaling cascade. Therapies that target OBCs have the advantage that they can re-activate early-stage processing of visual information within the inner retina. It is also at the level of the bipolar cells that incoming visual information is divided into ON- and OFF-pathways, which might be imperative for visual perception in higher visual centers (Thyagarajan et al., 2010). In mammals, the retina contains multiple ON- and OFF-types of cone bipolar cells and a single ON-type rod bipolar cell (RBC). Since the RBC signal diverges into cone ON- and OFF-pathways *via* the AII amacrine cell, optogenetic activation of OBCs restores responses in both, the ON- and OFF-pathways (Macé et al., 2015; van Wyk et al., 2015a).

Adeno-associated viral (AAV) vectors were proven to be powerful gene delivery vehicles to the photoreceptors and GCs; however, efficient transduction of the inner nuclear layer (INL), including the OBCs, has been a challenge. Novel AAV capsid variants generated by *in vivo* directed evolution in the mouse retina, including AAV7m8 (Dalkara et al., 2013), AAV8BP2 (Cronin et al., 2014) and Y-to-F mutations on the AAV2 capsid (Doroudchi et al., 2011) were shown to reach the inner retina. A short 200-bp *GRM6* enhancer sequence combined with the viral SV40 basal promoter is typically used to restrict optogene expression to OBCs (Kim et al., 2008). In order to increase transgene expression, Cronin and colleagues probed the efficacies of multiple copies of the 200-bp enhancer cassette and found optimal expression when using four enhancer elements in tandem (4x*GRM6*-SV40; Cronin et al., 2014).

While novel AAV variants perform well in mice, species-specific differences and individual differences in retinal degeneration can potentially result in different patterns of transgene expression. That is, an AAV vector that performs well in a wild-type mouse retina may not have the same efficacy in a degenerating retina or in the retina of a human. To address this concern, we evaluated the three most promising AAV capsid variants, AAV8BP2 (Cronin et al., 2014), AAV7m8 (Dalkara et al., 2013) and the single Y-F point mutant thereof, AAV7m8(Y444F) (Lu et al., 2016), side-by-side in two mouse models of photoreceptor degeneration—*rd1* and *rd10*—and in human retinal explants. The goal was to characterize the cellular expression patterns of the OBC optogenetic designer tool, Opto-mGluR6 (van Wyk et al., 2015a) after intravitreal AAV injection, which is the preferred application method in the clinic since it is less invasive than a subretinal injection and does not bear the risk of retinal tears or detachment.

We find that the 4x*GRM6*-SV40 promoter is *not* OBC specific, that all AAV variants possess a broad cellular transduction pattern in all systems, with differences between the AAV7m8 and AAVBP2 capsids as well as differences between the expression in murine and human retinas, and, most importantly, that all vectors target OBCs in healthy tissue but *not* in the degenerated *rd1* mouse model. Our study emphasizes the need to employ animal and human disease models for promoter and AAV development in light of a successful future clinical application.

## Results

In light of the ongoing efforts to develop an OBC-based gene therapy for optogenetic vision restoration, this study aimed to investigate side-by-side the OBC transduction efficacies of three state-of-the-art AAV capsid variants in healthy and degenerating mouse retinas and in human retinal explants. We equipped all viral vectors with the OBC-specific 4x*GRM6*-SV40 promoter (Cronin et al., 2014) and the Opto-mGluR6 designer optogene (van Wyk et al., 2015a) linked *via* an IRES sequence to the mCitrine fluorescent reporter gene.

### Cell-type specificity in the WT mouse retina

**Figure 1:**
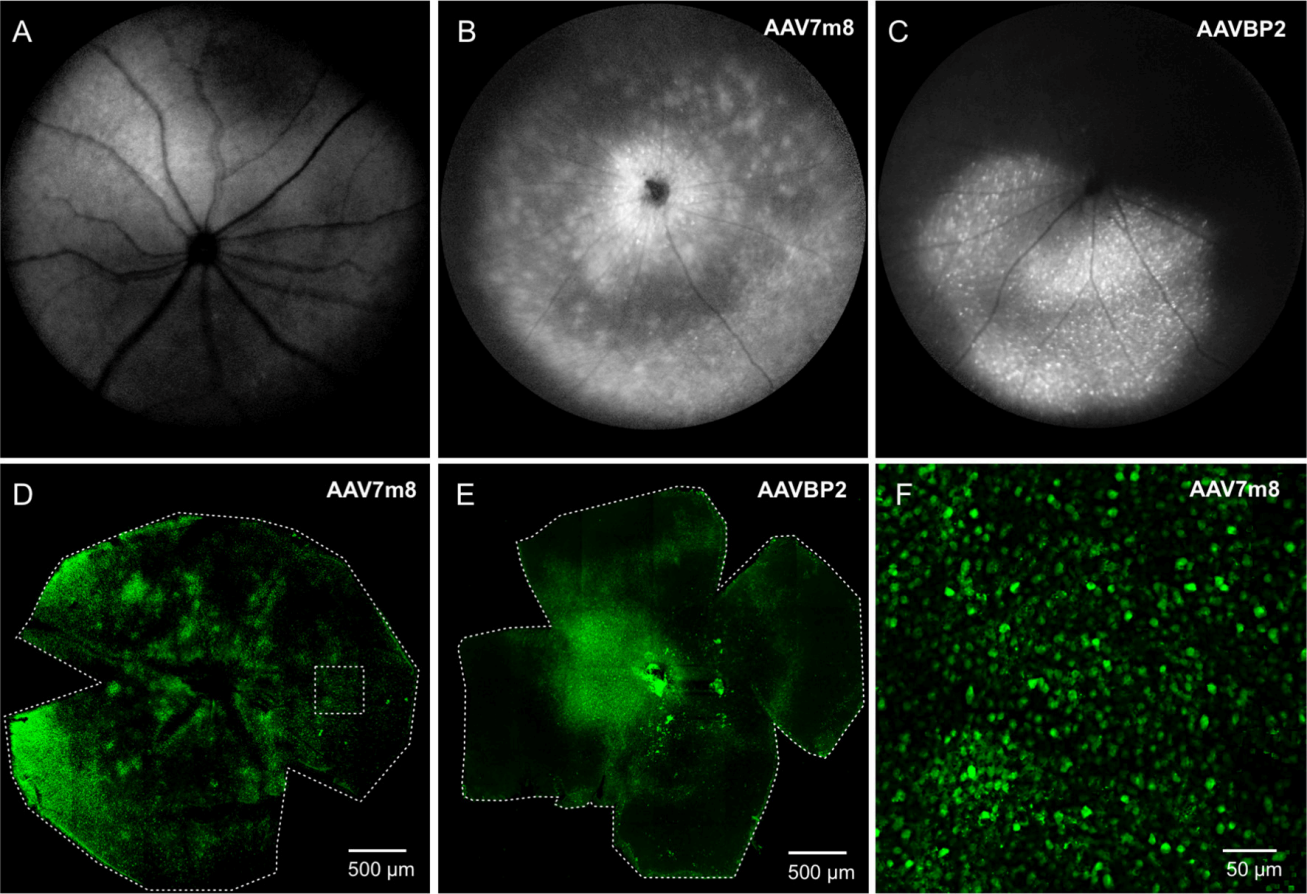
**Intravitreal injections of AAV7m8 and AAVBP2 into wild-type mice mediate panretinal expression of Opto-mGluR6_IRES_mCitrine under the 4x*GRM6*-SV40 promoter. (A–C)** Retinal *in-vivo* fundus fluorescence imaging of a control eye **(A)**, and eyes injected with AAV7m8 **(B)**, and AAVBP2 **(C)**, respectively, 3 weeks post injection. The retinas of the treated eyes show a large overall increase in fluorescence, with individual fluorescent cells often visible as bright specks. **(D,E)** Laser scanning micrographs of AAV7m8 **(D)** and AAVBP2 **(E)** treated retinal whole-mounts where mCitrine was labeled immunocytochemically. Despite more intense staining in some regions, mCitrine labeled cells are seen in all areas. **(F)** A higher magnification of the region indicated by the broken square in **(D)**.

**Figure 2:**
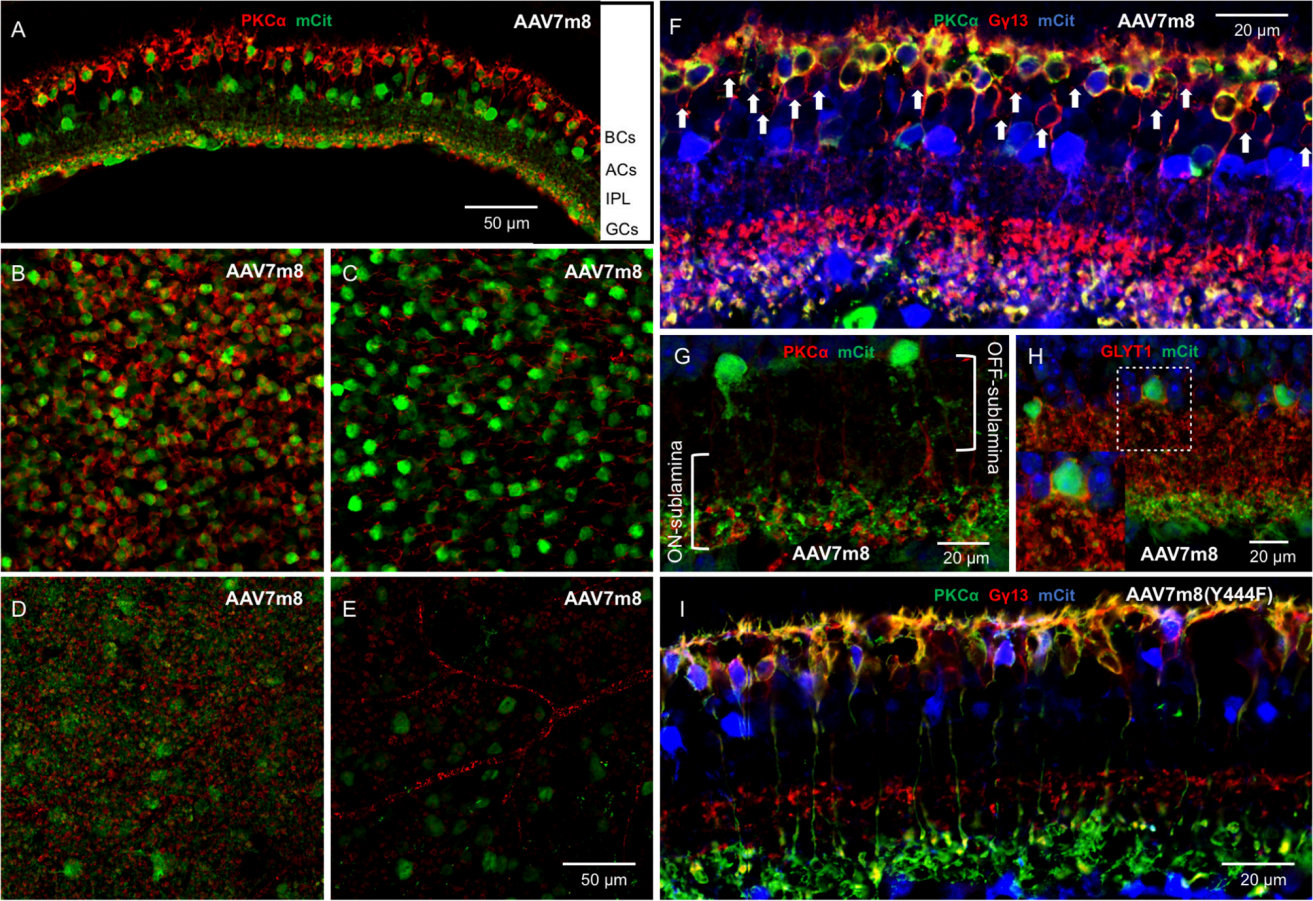
**Intravitreal injection of AAV7m8 in wild-type mice primarily expresses Opto-mGluR6_IRES_mCitrine in RBCs and AII amacrine cells when using the 4x*GRM6*-SV40 promoter. (A)** Transverse cryosection of an AAV7m8-treated retina with staining against PKCα (red, marker for RBCs) and mCitrine (green). Primarily two cell populations are labeled including the RBCs and a second layer of cell bodies in the amacrine cell (AC) layer. **(B–E)** Optical en-face sections of retinal whole-mounts labeled against PKCα and mCitrine taken at different depths from the same area using the same laser microscope settings. **(B)** Bipolar cell layer: most rod RBCs express mCitrine, with virtually no labeling in cells negative for PKCα. **(C)** Amacrine cell (AC) layer, a mosaic of brightly transduced cell bodies (green) and the axons of the RBCs (red) are seen. **(D)** Inner border of the inner plexiform layer (IPL), the fuzzy green background indicates the dendrites of the labeled ACs between the RBC terminals in red. **(E)** Weak mCitrine staining can be seen in some GCs. The secondary anti-mouse Cy3 antibody also labels blood vessels (red). **(F)** Triple labeling against mCitrine (blue), PKCα (green), and Gγ13 (red) confirms that transduced BPCs are largely RBCs (yellow) with nearly no labeling in cone OBCs that express Gγ13 but no PKCα (red; marked by arrowheads). **(G)** The labeled ACs (green) have the AII AC morphology with lobular processes in the OFF-sublamina of the IPL and more extensive dendritic arbors in the ON-sublamina including multiple close contacts with the axon terminals of PKCα-positive RBCs (red). **(H)** The amacrine cells expressing mCitrine (green) show GLYT1 reactivity (red) in the membranes of their cell bodies and their lobular processes in the OFF-sublamina. The insert shows a magnification of the broken square. **(I)** A micrograph with the same labeling as in **(F)** but treated with AAV7m8 (Y444F) instead of AAV7m8. The staining patter is similar, with mCitrine staining primarily restricted to RBCs and a single layer of ACs.

**Figure 3:**
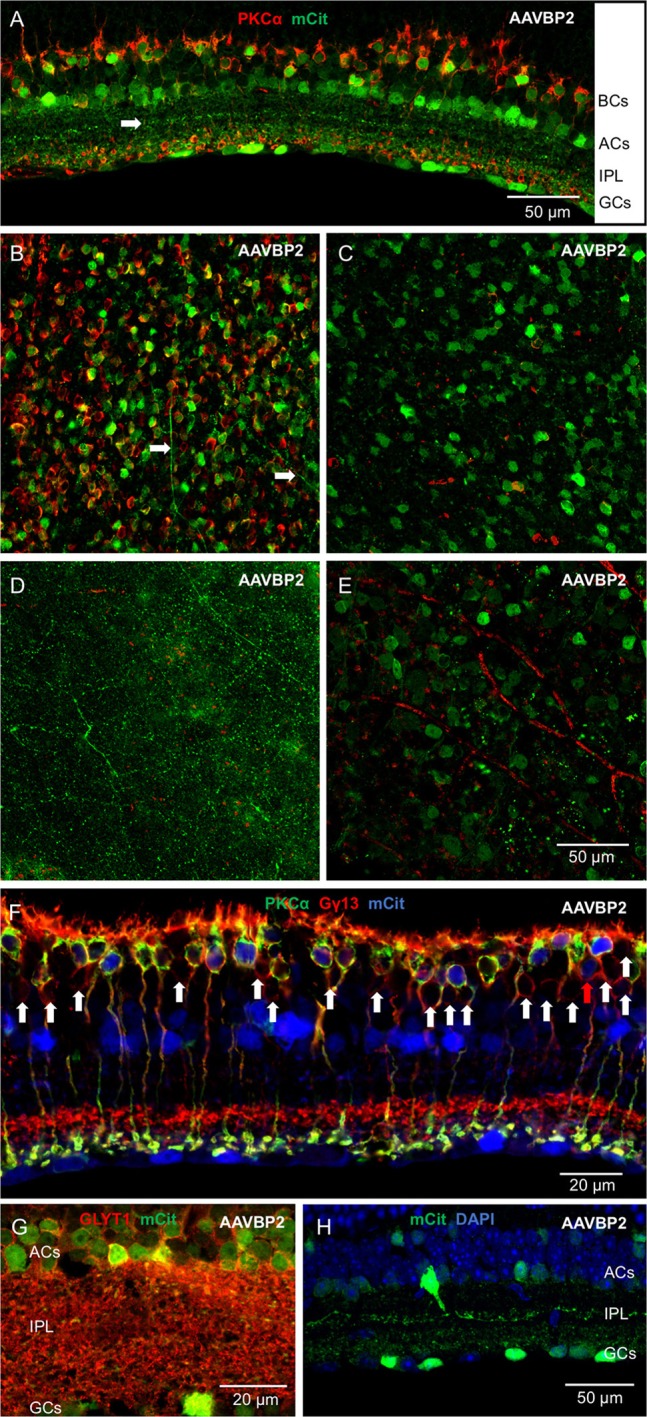
**Intravitreal injection of AAVBP2 in wild-type mice primarily expresses Opto-mGluR6_IRES_mCitrine in RBCs and several types of amacrine cells under the 4x*GRM6*-SV40 promoter. (A)** A cryosection labeled against PKCα (red) and mCitrine (green). Primarily two layers of cell bodies are labeled, RBCs and ACs with sparser labeling of GCs. The arrow shows a bright layer of dendrites in the middle of the IPL. **(B–E)** Optical en-face sections taken at different depths using the same laser microscope settings from the same area stained for PKCα (red) and mCitrine (green). **(B)** Bipolar cell layer: most RBCs express mCitrine. mCitrine labeling is, however, also seen in cell bodies not positive for PKCα. Horizontal cell processes can also be seen on this micrograph (arrows). **(C)** Amacrine cell layer: many cell bodies are labeled and the axons of RBCs are seen in red. **(D)** Middle of the IPL: a network of long beaded dendrites from wide-field amacrine cells is visible. **(E)** Weak mCitrine staining can be seen in some cell bodies in the GC layer. The secondary anti-mouse Cy3 antibody also labels the blood vessels (red). **(F)** Triple labeling against mCitrine (blue), PKCα (green), and Gγ13 (red) shows that transfected BPCs are largely RBCs (yellow) with nearly no labeling in cone OBCs that express Gγ13 but no PKCα (marked by arrow heads). The only weakly labeled cone OBC is indicated by a red arrow. **(G)** Most of the amacrine cells labeled in AAVBP2 retinas (green) express GLYT1 (red) in their cell membranes. **(H)** Example of a brightly labeled wide-field amacrine cell that projects long beaded dendrites to the center of the IPL (green).

**Figure 4:**
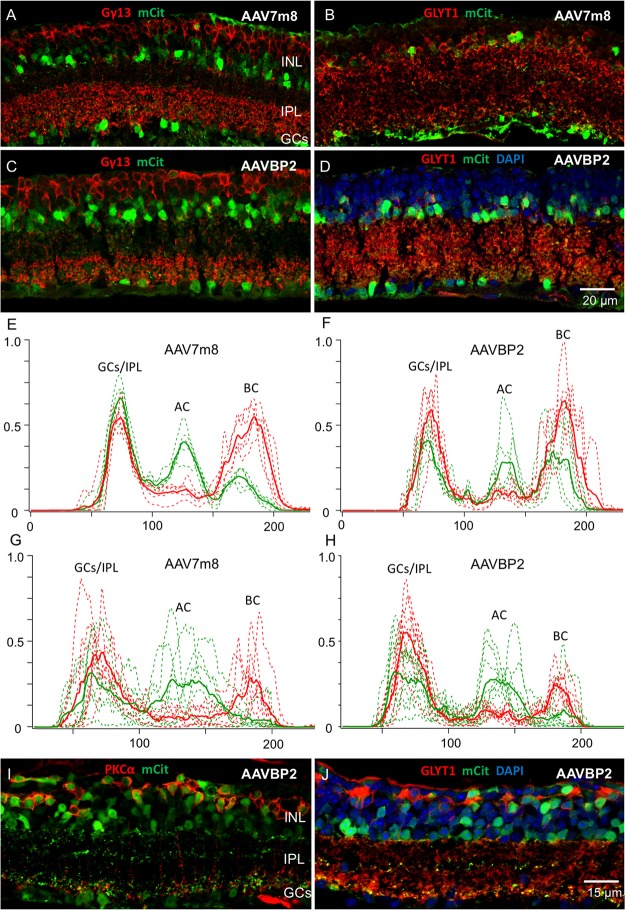
**AAV7m8 and AAVBP2 expression patterns in degenerating mouse lines. (A,B)** *rd1* mice injected with AAV7m8 at the age of 3.5 weeks show in hardly any mCitrine signal (green) in OBCs labeled with Gγ13 (**A**; red), while most mCitrine positive cells express GLYT1 (**B**; red). **(C,D)** As in **(A,B)**, *rd1* mice injected with AAVBP2 at the age of 3.5 weeks show little mCitrine expression in OBCs **(C)** compared to glycinergic amacrine cells **(D)**. **(E–H)** Intensity profiles of mCitrine expression in AAV7m8 **(E)** and AAVBP2 **(F)** treated wild-type retinas and in *rd1* AAV7m8 **(G)** and AAVBP2 **(H)** treated retinas. In **(E,F)** the mCitrine profiles (green) show three clear peaks in the GC/IPL, AC and BC layers. For reference, the PKCα signal is indicated in red, with two peaks indicating the cell bodies and axon terminals of the RBCs. Each figure shows the intensity profiles of six micrographs taken from three retinas (broken lines). The averaged signals are indicated in bold. **(G,H)** Analog intensity profiles of mCitrine expression in *rd1* retinas treated with AAV7m8 **(G)** and AAVBP2 **(H)**. The mCitrine signals in BCs are markedly reduced in the *rd1* retinas. **(I,J)** In the *rd10* retina AAVBP2 labels both, BPCs, and ACs similar to the wild-type retina. A large fraction of mCitrine-labeled cells (green) in the *rd10* retina express PKCα (**I**; red) and Goα (**J**; red).

We first assessed the overall abilities of AAV7m8 and AAVBP2 to mediate gene delivery to the wild-type C57/BL6 mouse retina. Intravitreal injection of both capsid variants resulted in strong pan-retinal mCitrine expression that was visible 3 weeks post-injection using *in vivo* fluorescence fundus imaging (Figures 1A–C). Although transduction was observed across the retina, we often found more extensive labeling around the optic nerve, in the peripheral retina and along large blood vessels. There were no overall quantitative or qualitative differences in the transgene expression patterns between retinas treated with AAV7m8 and AAVBP2 vectors that lie outside the variation observed between retinas within each treatment group (*n* ≥ 20 retinas per treatment group). To examine the cellular expression profiles in more detail, we labeled retinal wholemounts (Figures 1D–F) and vertical cryosections (Figures 2, 3) immunohistochemically against mCitrine. Despite the use of the “OBC-specific” 4x*GRM6*-SV40 promoter, mCitrine expression was *not* restricted to OBCs in retinas transfected with either of the AAV capsids. In addition to OBC labeling, treated retinas showed extensive mCitrine labeling in the amacrine cell layer, and weaker labeling in the GC layer (Figures 2, 3, 4E,F). In AAV7m8 treated retinas, we observed off-target expression of mCitrine in a single homogenous and evenly spaced amacrine cell type (Figures 2A,C). We identified these amacrine cells morphologically as AII amacrine cells based on their lobular processes in the OFF-sublamina of the IPL, their extensive dendritic arbors in the ON-sublamina including multiple close contacts with the axon terminals of PKCα-positive RBCs (Figure 2G) and their strong reactivity to an antibody against Glycine Transporter 1 (GLYT1; Figure 2H). Compared to AAV7m8, AAVBP2 labeled significantly more glycinergic amacrine cells (GLYT1-positive cells; *P* = 0.0045; Table 1), which appear morphologically different (Figures 3A,C,G). AAVBP2 also labeled a bright plexus of wide-field amacrine cells with long beaded dendrites that stratify in the center of the IPL (Figures 3A,D,H) and occasionally, B-Type horizontal cells (Figure S1).

**Table 1:**
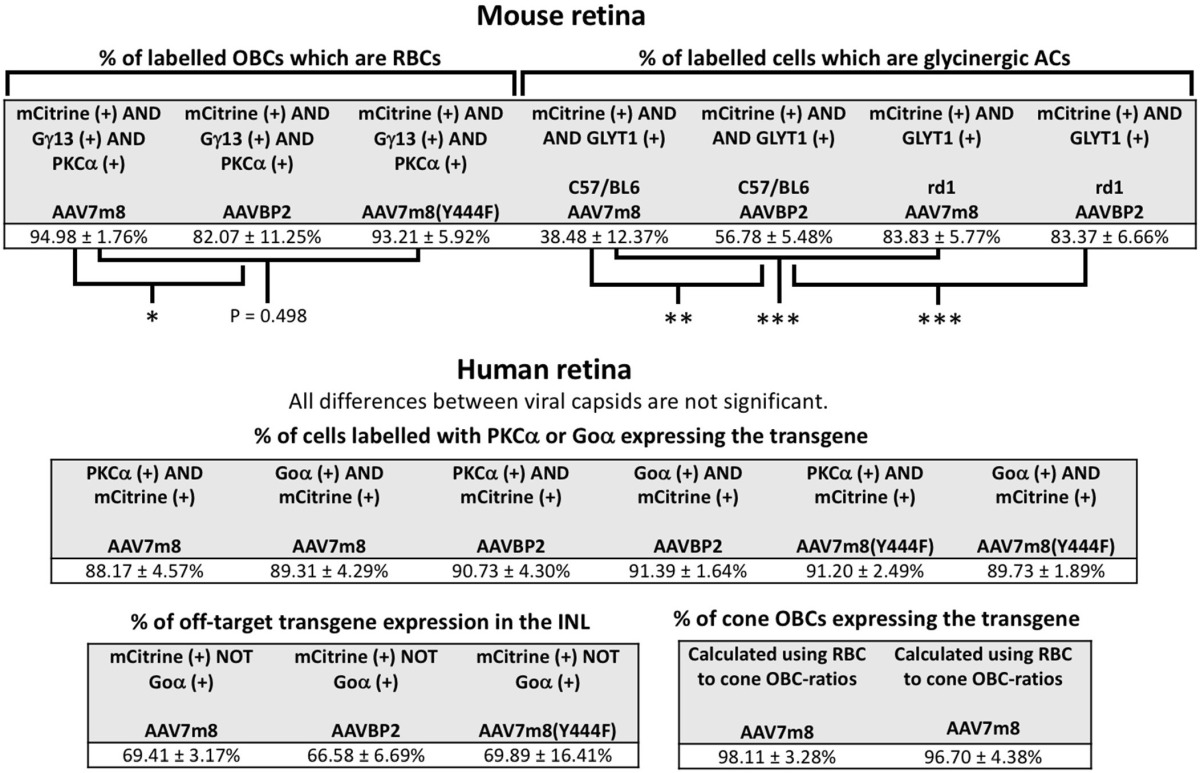
**Cell counts in mouse and human retinas**.

To identify the transduced bipolar cell types, we performed triple staining against mCitrine, Gγ13 (labels all OBCs) and PKCα (specific for RBCs). Although the 4x*GRM6*-SV40 promoter was expected to equally mediate expression in cone OBCs and in RBCs, the AAV7m8 capsid almost exclusively labeled RBCs (95.0 ± 1.8% RBCs vs. 5.0 ± 1.8% cone OBCs; Table 1; Figure 2F). AAVBP2 transduced significantly more cone OBCs compared to AAV7m8 (*P* = 0.02), however, expressing cells were still predominantly RBCs (82.1 ± 11.3 % RBCs vs. 17.9 ± 11.3% cone OBCs; Table 1; Figure 3F).

Since it was shown that the Y444F point mutation on the AAV7m8 capsid improves the overall transgene expression and also enables expression in cone OBCs (Lu et al., 2016), we probed mCitrine expression when using the AAV7m8(Y444F) capsid (*n* = 6 eyes). In our hands AAV7m8(Y444F) did not enhance expression nor did it cause any changes in cellular tropism compared to AAV7m8, with no significant difference in the percentage of mCitrine-labeled OBCs that were also PKCα positive (*P* = 0.5; Table 1; Figure 2I). Again, expression was almost exclusively restricted to RBCs and AII amacrine cells with some GC labeling. Since we observed no effective differences between the AAV7m8 and the AAV7m8(Y444F) capsids, we did not further investigate the tropism of AAV7m8(Y444F) in the mouse retina.

### Cellular tropism in the degenerating mouse retina

Multiple studies have described morphological and physiological changes in the inner retina after photoreceptor degeneration. Some of these changes include a retraction of bipolar cell dendrites and synaptic rewiring (Strettoi and Pignatelli, 2000; Jones et al., 2012), the generation of rhythmic oscillations in the inner retina (Menzler and Zeck, 2011) and changes in protein expression and trafficking (Puthussery et al., 2009; Xu et al., 2012). It is therefore not justified to assume that the activities of promoters and AAV capsids remain unaffected when the photoreceptors degenerate. Accordingly, we next investigated the cellular transduction patterns of AAV7m8 and AAVBP2 in the most commonly used mouse model of retinal degeneration, the Pde6b^rd1^ (*rd1*) mouse (Chang et al., 2002; van Wyk et al., 2015b). In the *rd1* mouse, degeneration starts with a fast onset of rod dystrophy at postnatal day (P) 8–10, followed by a peak in rod degeneration at P14 and a near complete loss of rods and RBC dendrites by P21. Degeneration of the rod system is marked by a total loss of scotopic ERG responses in the 4^*th*^ week of life. A secondary degeneration of cones starts in the 5^th^ week of life and the dendrites of the cone bipolar cells retract. The main peak of cone degeneration is in the 7^th^–8^th^ week of life; however, a single row of cone photoreceptors persists up to the age of 6 months (Farber et al., 1994; Hackam et al., 2004; van Wyk et al., 2015b).

In a first round we injected strongly degenerated, 4-month-old *rd1* mice intravitreally with each capsid type (*n* = 8 eyes per capsid). Similar to wild-type retinas, we removed *rd1* retinas after a 4-week viral incubation period and probed mCitrine expression. Surprisingly, while the labeling of glycinergic amacrine cells remained unaffected in these *rd1* retinas, the mCitrine signal in RBCs was nearly completely abolished (Figures 4G,H). We also observed no labeling of wide-field amacrine cells or B-Type horizontal cells in AAVBP2 treated *rd1* retinas. These results suggest substantial changes in the gene expression profile of the degenerating retina.

As both, AAV7m8 and AAVBP2 vectors almost exclusively target RBCs, which remodel within the 4^th^ postnatal week and in this process potentially downregulate expression from the *GRM6* enhancer (Puthussery et al., 2009), we additionally injected a second group of *rd1* mice shortly after weaning (3.5 weeks old; *n* = 6 eyes per capsid). The transduction patterns in these younger *rd1* mice remained, however, identical to older *rd1* mice with lacking expression in OBCs. The loss of OBC labeling in *rd1* retinas resulted in a significantly higher fraction of labeled cells expressing GLYT1 both in AAV7m8 (*P* < 0.0001) and AAVBP2 (*P* < 0.0001) treated *rd1* retinas compared to wild-type retinas (Figures 4A–D; Table 1; *n* ≥ 6 micrographs collected from *n* ≥ 4 retinas).

To investigate this phenomenon in more detail, we probed AAVBP2-mediated expression in an alternative, slower degenerating mouse model, the Pde6b^rd10^ (*rd10)* mouse. The *rd10* mouse has a missense mutation in exon 13 of the same *Pde6b* gene affected in *rd1* mice resulting in partial Pde6b activity. Compared to *rd1* mice, the onset of degeneration is later at P18 and the progression slower (Gargini et al., 2007). The scotopic ERG is lost in postnatal week 8, indicating complete loss of rods and remodeling of RBCs (Gargini et al., 2007; van Wyk et al., 2015b). We therefore injected 11-week-old *rd10* mice (*n* = 6 eyes) with a degenerative state approximately corresponding to 4-week-old *rd1* mice. Unexpectedly, the cellular expression pattern in *rd10* mice was similar to that observed in wild-type C57/BL6 retinas, with high levels of RBC labeling (Figures 4I,J). It is important to note that, after the viral incubation period of 4 weeks, degeneration in the now 8-week-old *rd1* retinas might be more severe compared to the *rd10* mice, which we sacrificed at the age of 15 weeks. The results may imply that 4x*GRM6*-SV40 promoter driven gene expression is downregulated in a phenotype dependent manner.

### Cellular tropism in the human post-mortem retinal explant

Due to numerous anatomic and physiologic differences between the eyes of mice and “men,” we evaluated the transduction characteristics of all three capsids, AAV7m8, AAV7m8(Y444F), and AAVBP2 in post-mortem human retinal explants. We prepared and treated explants from a total of five donor eyes. After 7 days in culture, a homogenous mCitrine expression became clearly visible, with some enhanced expression at the edges of the explants, around blood vessels as well as in damaged areas, likely due to better tissue access of the AAVs. Besides some thinning of the retinal layers the explants remained well preserved during culturing.

**Figure 5:**
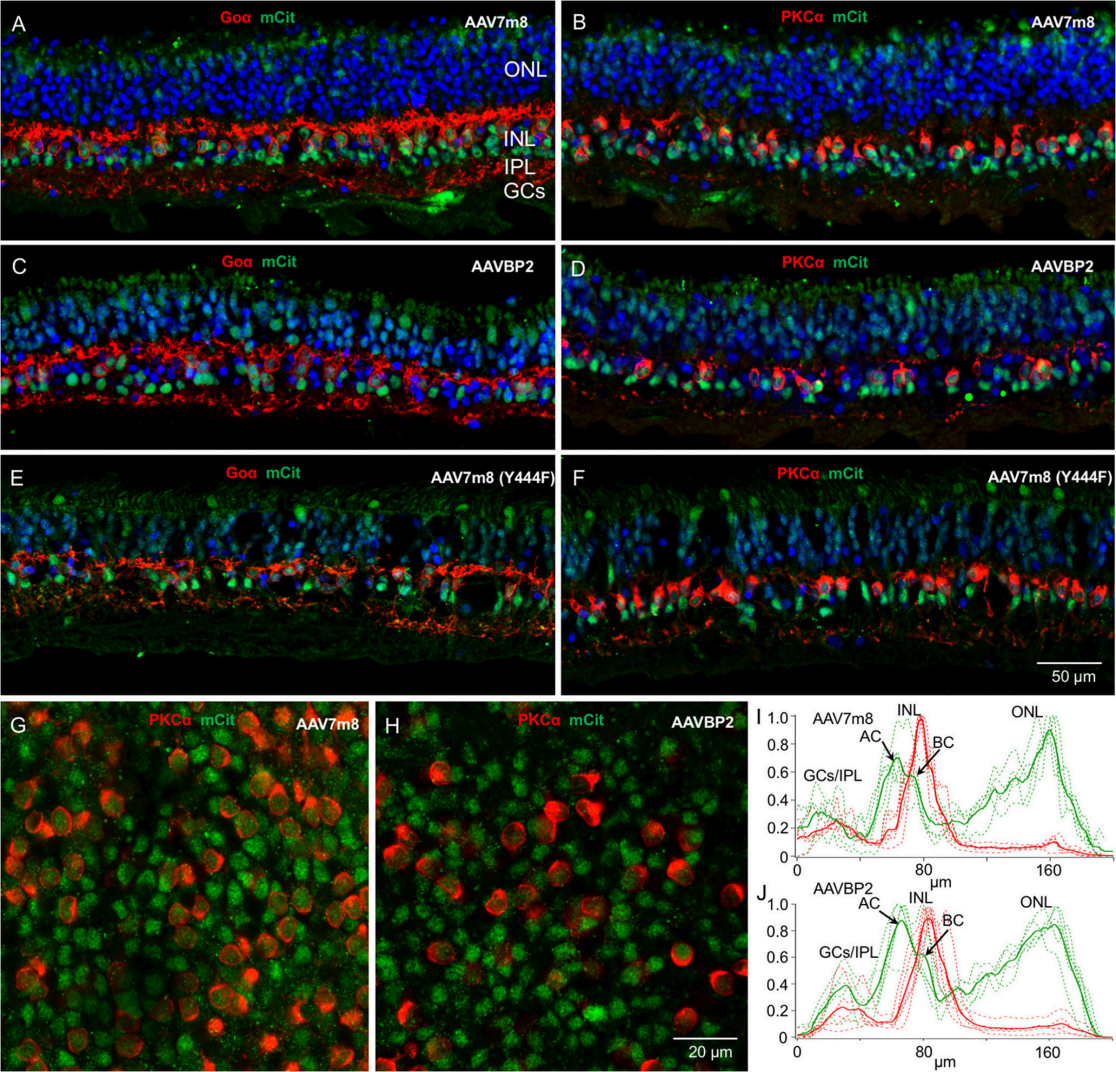
**Transduction patterns of AAV7m8, AAVBP2, and AAV7m8 (Y444F) in human retinal explants. (A–F)** Vertical cryosections of human retinal explants transduced with AAV7m8 **(A,B)**, AAVBP2 **(C,D)**, and AAV7m8 (Y444F) **(E,F)** were labeled with antibodies against mCitrine (green) and Goα (**A,C,E**, red) or PKCα (**B,D,F**, red). A similar expression pattern is observed in retinas treated with all three capsids: Opto-mGluR6_IRES_mCitrine is preferentially expressed in the INL, with high expression in OBCs (Goα-positive cells) but also in Goα-negative cells. **(G,H)** Whole-mount images at the level of the INL of explants treated with AAV7m8 **(G)** and AAVBP2 **(H)** stained for mCitrine (green) and PKCα (red). **(I,J)** Intensity profiles of mCitrine expression in AAV7m8 **(I)** and AAVBP2 **(J)** treated explants. For reference, the PKCα signal is indicated in red. mCitrine profiles (green) show a similar distribution, with a large peak in the INL and a second large peak in the ONL which gradually increases toward the outer ONL. A double peak in the INL shows labeling both in the AC layer (first peak) and in the BC layer (second peak). Each figure shows the intensity profiles of 4 images taken from immune-labeled transverse cryosections of two explants (broken lines). The averaged signals are indicated in bold.

**Figure 6:**
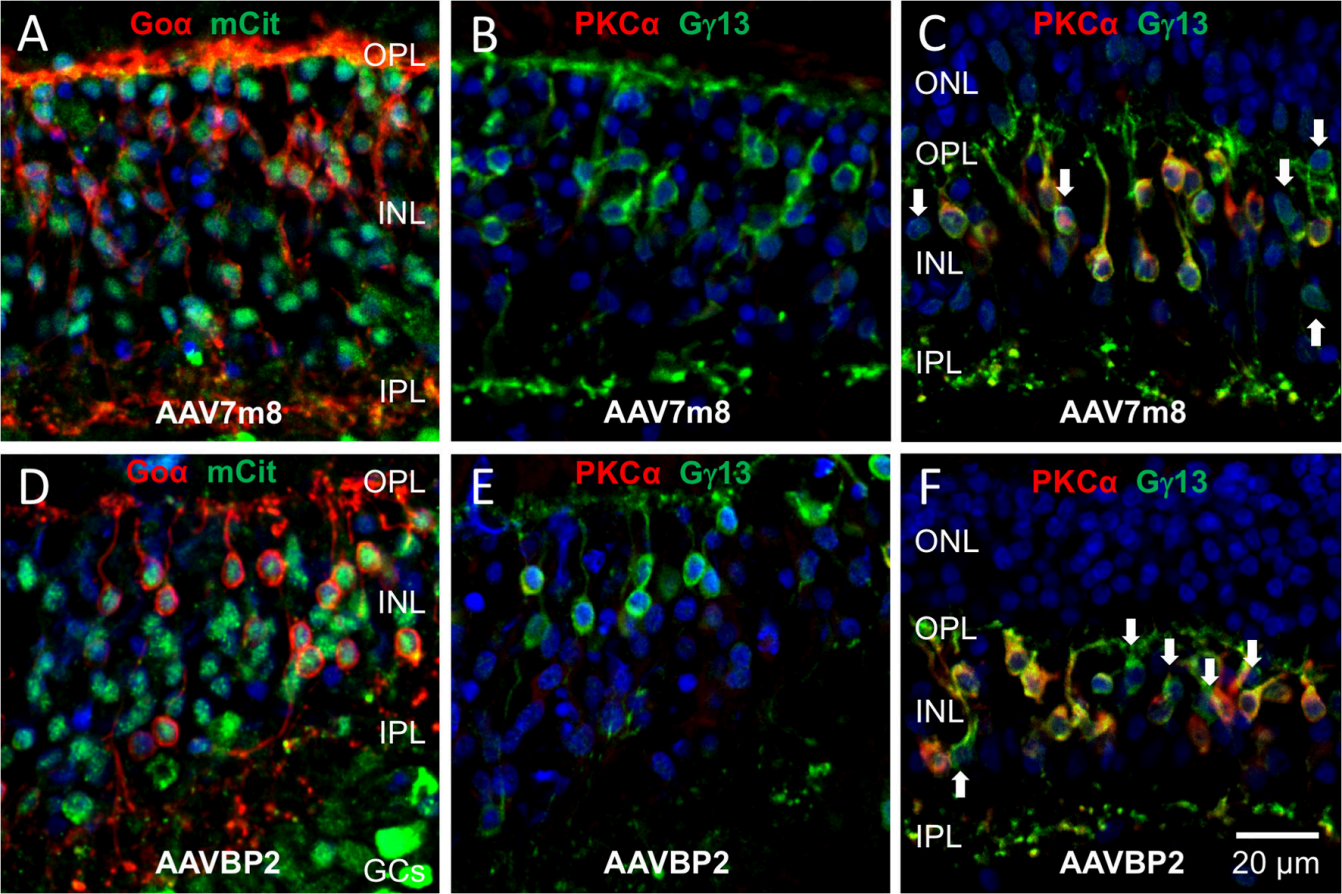
**Cone OBCs near the fovea centralis express the transgene in human retinal explants transduced with AAV7m8 and AAVBP2. (A–F)** Vertical cryosections of human retinal explants transduced with AAV7m8 **(A–C)** or AAVBP2 **(D–F)**. **(A,D)** Labeling against Goα (red) and mCitrine (green) near the fovea centralis shows transgene expression in OBCs. **(B,E)** Labeling against PKCα (red) and Gγ13 (green) of the same regions of neighboring sections to **(A)** and **(D)** show that no cells in this region were labeled by PKCα, confirming their cone OBC identity. **(C,F)** In comparison to (**B,E)**, sections from the mid-periphery show a high fraction of Gγ13-positive cells (green), which also express PKCα (red). OBCs not positive for PKCα are indicated by arrows.

Similar to the mouse retina, the 4x*GRM6*-SV40 promoter was not OBC-specific and led to broad transgene expression throughout the explants (Figure 5). There were some clear differences in the cellular transduction patterns in human tissue compared to the mouse retina: Cone OBCs were transduced equally well as RBCs (Figure 6; Table 1), a significant number of photoreceptors also expressed the transgene and the three capsids showed identical cellular tropism (Figure 5). About 90% of Goα-positive cells expressed the transgene, independent of the AAV capsid used (Table 1). The same percentage values were found for transgene expressing PKCα-positive cells (Table 1), suggesting that RBCs and cone OBCs were targeted equally well by all capsids. Since viral incubation was limited to 7 days and mCitrine was expressed behind an IRES motif designed for low expression (cloned from the pIRES2 plasmid), only one of our mCitrine antibodies gave strong enough staining for evaluation in human explants. As this antibody was raised in a rabbit host, we were not able to triple label mCitrine, PKCα and Gγ13/Goα (see Antibody table). Therefore, staining against Gγ13 and PKCα were used to determine the fraction of RBCs within the OBCs population in individual explants, which ranged from 78% to 90%. These fractions were then used to estimate the percentage of transgene expressing cone OBCs in the respective explant. On average, this percentage was calculated to be ~97% for both, AAV7m8 and AAVBP2-transduced explants (*n* = 3 retinas per capsid variant; Table 1). To further verify high expression levels in cone OBCs, we also transduced explants from the fovea centralis, which is virtually devoid of rods and RBCs. Again, we observed high numbers of transfected OBCs, confirming good transduction of cone OBC with AAV7m8 and AAVBP2 in the human retina (Figure 6).

In all explants, independent of the capsid used, we observed off-target transgene expression in photoreceptor cells, in some GCs and in Goα-negative cells, presumably amacrine cells, in the INL (Figure 5). For all capsids, ~69% of cells labeled in the INL were not OBCs (*n* = 2 retinas per capsid variant; Table 1).

## Discussion

Retinal OBCs make attractive targets for optogenetic vision restoration. Potential therapies targeting OBCs demand efficient and specific optogene delivery and expression. Here, we examined the abilities of the newly engineered AAV capsid variants AAV7m8, AAVBP2, and AAV7m8(Y444F) in combination with the state-of-the-art “OBC specific” 4x*GRM6*-SV40 promoter (Kim et al., 2008; Cronin et al., 2014; van Wyk et al., 2015a) to deliver the optogenetic designer tool Opto-mGluR6 to retinal OBCs. Due to the known anatomic and physiologic differences between healthy and degenerating retinas and between the retinas of mice and humans, we compared reporter expression patterns in both healthy and degenerating mouse retinas and in *post-mortem* human retinal explants.

We first tested the capsids side-by-side in the wild-type C57/BL6 mouse retina. The three capsid variants penetrated equally well to the INL. However, despite the identical enhancer-promoter used in all capsids, AAVBP2 and AAV7m8 showed a different cell-type specific transduction pattern. Although both capsids had a preference for RBCs and amacrine cells, AAVBP2 showed a wider tropism. A preference of AAV7m8(Y444F) for RBCs over cone OBCs was previously shown (Lu et al., 2016). In our hands, AAV7m8 and AAV7m8(Y444F) showed a virtually identical, non-specific transduction pattern. All viruses mediated significant off-target labeling in the mouse and human retinas, particularly of glycinergic amacrine cells and, most strikingly, none of the viruses were able to transduce OBCs in the degenerated *rd1* retina.

As the AAV capsids were titer-matched to the minimal dose that efficiently transduces cells in the INL (Cronin et al., 2014), we conclude that the 4x*GRM6*-SV40 promoter, and consequently also the *GRM6*-SV40 promoter, are not “OBC specific” promoters as commonly believed. Recently, an endogenous murine *Grm6* promoter variant has been developed that was reported to reduce off-target expression compared to the *GRM6*-SV40 promoter (Lu et al., 2016). The non-specificity of the 4x*GRM6*-SV40 promoter, however, allowed us to differentiate between the cellular transfection patterns of each capsid variant and most importantly, it allowed us to compare OBC transfection in wild-type and degenerating retinas using off-target expression as an internal reference.

We showed for the first time a clear qualitative difference in AAV-driven expression in the commonly used *rd1* mouse model of rod dystrophy compared to the wild-type mouse. In all *rd1* retinas transduced with AAV7m8 or AAVBP2, the OBCs showed hardly any reporter expression. Since we still observed OBC expression in *rd10* retinas, we hypothesize that the 4x*GRM6*-SV40 promoter is downregulated in late stages of photoreceptor degeneration. Since the cones are lost in a secondary stage of degeneration and cone OBCs were shown to remain responsive to glutamate until ~P180 (Puthussery et al., 2009), AAV variants that have the ability to transduce cone OBCs are favored when treating rod dystrophy. Likewise, promoters based on alternative OBC-specific genes, which are not downregulated during retinal degeneration, might be preferable. These considerations have to be taken into account when developing a therapy aimed at restoring vision in patients suffering from photoreceptor degeneration.

Previous studies, however, were able to record light responses from GCs in the *rd1* retina treated with AAV-based gene therapies, using both the 1x and 4x*GRM6*-SV40 promoters (Cronin et al., 2014; Macé et al., 2015; van Wyk et al., 2015a). One possible explanation for functional recovery is that low-level optogene expression in OBCs suffices to drive weak light responses in *rd1* retinas. As we show here, OBC labeling in the *rd1* retina is strongly downregulated but not completely absent. Also, we cannot exclude the possibility that light responses were, at least in part, driven by off-target cells. One of the studies, however, demonstrated OBC-specific ChR2 expression anatomically in *rd1* mice of similar age as used in this study (Macé et al., 2015). The basis for this discrepancy is not obvious. One possibility is phenotype-dependent downregulation of the *GRM6*-SV40 promoter with progressing degeneration. We have previously demonstrated that secondary genetic factors affect the phenotype of degeneration in different *rd1* strains (van Wyk et al., 2015b), potentially also affecting the activity of the *GRM6*-SV40 promoter; Macé and colleagues used the C3H/HeN *rd1* mouse line, whereas we used the C3H/HeOu line. The 1x*GRM6*-SV40 promoter has also been used to introduce ChR2 into the OBCs of the *rd1* retina by electroporation of new-born mouse pups (Lagali et al., 2008). It is difficult, however, to relate the expression achieved with electroporation of neonatal mice with that of an AAV-based gene therapy in adult mice.

For a more clinically relevant comparison we also tested the transduction patterns in *post-mortem* human retinal explants. In these experiments transduction was wide-spread and similar for all capsid variants contrasting previous studies that reported successful transduction of human explants only in the parafoveal region and along blood vessels (Sengupta et al., 2016). In line with the fact that the morphology of the eyes of mouse and human differ, the overall cell transduction pattern differed, with marked expression in the photoreceptor cells of human explants. Remarkably, the cellular transduction pattern was virtually identical to that reported for the unspecific CMV promoter (Cronin et al., 2014).

We found no overall quantitative difference between AAV7m8, AAVBP2, and AAV7m8(Y444F) mediated transgene expression in the human retina. The high levels of transduction, including off-target photoreceptor labeling, may be attributed to the increased “viral pressure,” as AAV solutions were directly applied to the explants allowing optimal access of the virus to the cells. The distinct differences between the expression patterns in human retinal explants and the murine retina show that the actions of the viral vectors and/or the 4x*GRM6*-SV40 promoter are species-dependent.

Off-target expression using a “leaky” promoter such as 4x*GRM6*-SV40 may not always be a disadvantage. In general, there exist two therapeutic approaches that target OBCs, the first using ChR2 or variants thereof (Lagali et al., 2008; Macé et al., 2015) and a second using Gi-coupled photopigments that feed into the native mGluR6 signaling cascade of the OBCs, rendering their function OBC specific (Cehajic-Kapetanovic et al., 2015; van Wyk et al., 2015a). In the mouse, AAV7m8 almost exclusively transduces two cell types, the RBCs and the AII amacrine cells, making it a suitable tool for either approach. When using ChR2, the light-activated depolarizing drive of the RBCs is relayed to the AII amacrine cells that will now be equally depolarized, potentially increasing the drive of the cone ON- and OFF-bipolar cells. Additional transduction of GCs, although relatively weak, will perturb the visual signal when ChR2 variants are used since all GCs that express ChR2 will depolarize in response to light, corrupting the OFF-channel. This may not be a problem when Gi-coupled optogenetic tools are used, as GCs are less likely to generate a large response due to a lack of matching intracellular signaling components. The extensive tropism of AAVBP2, including the transduction of wide-field amacrine cells and horizontal cells, may compromise visual resolution when combined with a ChR2 variant. Again, for Gi-coupled optogenetic tools this may not be an issue.

Our study emphasizes the need to employ animal and human disease models when advancing of optogenetic treatment strategies targeted at the inner retina.

## Materials and methods

### AAV vectors

The OBC-specific mouse 4x*GRM6* enhancer sequence (see below) in combination with the viral SV40 basal promoter (Cronin et al., 2014) was PCR amplified (F: 5′-TAT AGC TAG CAC GCG TGA TCT CCA GAT GGC TAA AC, R: 5′-TAT AAG ATC TAA GCT TTA TAG GAT CCG GTA CCT TTG CAA AAG CCT AGG CC) from the pAAV-*4xGRM6*-CatCh-EGFP plasmid (received from B. Roska under an MTA by the Friedrich Miescher Institute for Biomedical Research, Basel, Switzerland), cut with BglII and NheI and inserted into a linearized pAAV-Rho-EGFP vector (kind gift from A. Auricchio) (Allocca et al., 2007) that was cut with BamHI and NheI (BamHI and BglII have compatible overhangs) to create the pAAV-4x*GRM6*-SV40 plasmid. Opto-mGluR6_IRES_mCitrine (van Wyk et al., 2015a) was inserted between the HindIII and BamHI restriction sites of the pAAV-4x*GRM6*-SV40 plasmid using an In-Fusion 2.0 homologous recombination Kit (Clontech). Viral vectors were produced in AAV-293 cells by triple plasmid co-transfection. We co-transfected the pAAV-4x*GRM6*-SV40_Opto-mGluR6_IRES_mCitrine expression plasmid, the AAV-helper plasmid encoding Rep2 and Cap for serotype variants, and the pXX680 plasmid harboring helper adenoviral genes (kindly provided by H. Büning) using the calcium-phosphate precipitation method. Empty virions were removed by density purification over an iodixanol gradient (Axis-Shield, Oslo) and the 40% iodixanol fraction subsequently buffer exchanged by amicon filtration (Millipore). The AAV fraction was titered for DNase-resistant vector genomes by real-time PCR relative to a standard vector. The AAV7m8 capsid plasmid was a kind gift from D. Dalkara and the BP2 capsid plasmid was a kind gift from T. Cronin. The AAV7m8(Y444F) capsid plasmid was generated by site-directed point mutagenesis (QuikChange kit) on the AAV7m8 Rep2-Cap plasmid. Vectors were stored at −80°C until just before use. To allow for a direct comparison, we titer-matched all AAV variants in sterile PBS to 1.8 × 10^12^ genome copies ml^−1^ before injection.

#### Complete sequence of the 4x*GRM6*-SV40 promoter

ACGCGTGATC TCCAGATGGC TAAACTTTTA AATCATGAAT GAAGTAGATA TTACCAAATT GCTTTTTCAG CATCCATTTA GATAATCATG TTTTTTGCCT TTAATCTGTT AATGTAGTGA ATTACAGAAA TACATTTCCT AAATCATTAC ATCCCCCAAA TCGTTAATCT GCTAAAGTAC ATCTCTGGCT CAAACAAGAC TGGTTGCTCG ACATTGATTA TTGACTAGTG ATCTCCAGAT GGCTAAACTT TTAAATCATG AATGAAGTAG ATATTACCAA ATTGCTTTTT CAGCATCCAT TTAGATAATC ATGTTTTTTG CCTTTAATCT GTTAATGTAG TGAATTACAG AAATACATTT CCTAAATCAT TACATCCCCC AAATCGTTAA TCTGCTAAAG TACATCTCTG GCTCAAACAA GACTGGTTGC TCGACATTGA TTATTGACTA GTGATCTCCA GATGGCTAAA CTTTTAAATC ATGAATGAAG TAGATATTAC CAAATTGCTT TTTCAGCATC CATTTAGATA ATCATGTTTT TTGCCTTTAA TCTGTTAATG TAGTGAATTA CAGAAATACA TTTCCTAAAT CATTACATCC CCCAAATCGT TAATCTGCTA AAGTACATCT CTGGCTCAAA CAAGACTGGT TGCTCGACAT TGATTATTGA CTAGTGATCT CCAGATGGCT AAACTTTTAA ATCATGAATG AAGTAGATAT TACCAAATTG CTTTTTCAGC ATCCATTTAG ATAATCATGT TTTTTGCCTT TAATCTGTTA ATGTAGTGAA TTACAGAAAT ACATTTCCTA AATCATTACA TCCCCCAAAT CGTTAATCTG CTAAAGTACA TCTCTGGCTC AAACAAGACT GGTTGCTCGA GATCTGCGAT CT**GCATCTCA ATTAGTCAGC AACCATAGTC CCGCCCCTAA CTCCGCCCAT CCCGCCCCTA ACTCCGCCCA GTTCCGCCCA TTCTCCGCCC CATCGCTGAC TAATTTTTTT TATTTATGCA GAGGCCGAGG CCGCCTCGGC CTCTGAGCTA TTCCAGAAGT AGTGAGGAGG CTTTTTTGGA GGCCTAGGCT TTTGCAAAAA GCTT**

Gray highlights indicate four tandem repeats of the enhancer sequence and “bold letters” indicate the SV40 promoter.

### Animals and injections

We obtained C57Bl/6J from breeding stock at the Jackson Laboratory (Bar Harbor, ME) and C3H/HeOu (*rd1*) and B6.CXB1-Pde6brd10/J (*rd10*) mice from breeding stocks at the Charles River Laboratories (Sulzfeld, Germany). All animal experiments and procedures were reviewed and approved by the Swiss Veterinary Office. All injections were performed by the same skilled person. Wild type mice were injected at an age between 4 and 6 weeks, the age of injection of the different degenerating treatment groups are specified in the Results Section: *rd1* mice were injected at 3.5 and 16 weeks of age, *rd10* mice at 11 weeks of age. Mice were anesthetized by intraperitoneal injection of 100 mg/kg ketamine and 10 mg/kg xylazine. We then punctured the sclera on the nasal side ~1 mm from the corneal limbus using a 30G needle. The puncture needle was removed, and a 33G blunt needle was maneuvred through the hole to the back of the eye (RPE injection kit from World Precision Instruments). We then injected 2 μl of titer-matched virus (1.8 × 10^12^ GC ml^−1^) solution into the vitreous and waited for 20 s before retracting the injection needle form the eye. Following surgery, the eyelids were stuck closed using petroleum jelly to prevent drying of the cornea.

### Human post-mortem retinal explants

All procedures were in compliance with governmental regulations and in accordance with the tenets of the Declaration of Helsinki. The Swiss Ethics Committee has reviewed this study and exempted it from the ethics review process. As per national laws and regulations [Federal Act on Research involving Human Beings (Human Research Act, HRA 810.30, Art. 38)], no ethics approval is required for this type of study. Anonymized tissue was provided by the Department of Ophthalmology, Inselspital, Bern University Hospital, Switzerland. Preparation of human retinal explants was performed as previously described (Fradot et al., 2011). After removal of the cornea, eyes were stored in cold phosphate-buffered saline (PBS). Post-mortem delay times for explanting never exceeded 20 h. Immediately after receiving the eyes, the retina was isolated and transferred to HEPES buffered Ames' medium containing Gentamicin (50 μg/mL; Arman and Sampath, 2010). Retinal fragments of ~0.5 cm^2^ were prepared from the mid-periphery of the retina (8–15 mm distal from the macula) and the macula (see Figure S2) and transferred with the GC layer facing up on to Millicell hanging cell culture inserts (12-well, PET, 0.4 μm, Merck Millipore, PIHT15R48). The explants were incubated in R16 medium with supplements (Romijn, 1988) for 2 h prior to AAV administration. We placed 5 μl of AAV solution (1.8 × 10^12^ GC ml^−1^) directly on top of the explants. After 1 day, 200 μl of fresh medium was added to each culture followed by a daily exchange of 350 μl of medium until fixation on day 7 of culturing.

### *In vivo* fundus imaging of mouse retinas

Transduction was assessed *in vivo* by recording the mCitrine signal using confocal scanning laser ophthalmoscopy (Spectralis OCT, Heidelberg Engineering GmbH, Heidelberg, Germany). Imaging in anaesthetized mice was performed in blue light (488 nm) autofluorescence mode using a non-contact ultra-widefield 102° lens. The mice were placed on a custom-made platform for optimal alignment of the eye with the optical axis of the imaging device, and corneas were protected from desiccation with hydroxypropylmethylcellulose (Methocel 2%, OmniVision, Neuhausen, Switzerland). The large vessels were brought into focus in near infrared mode, and a slight further adjustment to re-focus was made after switching to autofluorescence mode to compensate for wave-length dependent differences in refraction.

### Immunohistochemistry

We removed mouse retinas 4 weeks post-injection for immunohistochemical analysis. Mouse eyes and human retinal explants were fixed with 4% (wt/vol) paraformaldehyde in PBS for 40 min at room temperature (RT), cryoprotected over three consecutive nights at 4°C in graded sucrose solutions (10%, 20%, and 30% sucrose in PBS), embedded in cryomolds with O.C.T. compound (Sakura Finetek), and frozen in liquid nitrogen-cooled 2-methylbutane. Vertical sections of 10 μm thickness were cut on a cryostat, mounted on SuperFrost glass slides (Menzel) and stored at −20°C until use. O.C.T. was removed by placing slides in PBS for 5 min. The sections were subsequently lined with an oil pen and covered in blocking solution (6% vol:vol normal goat or donkey serum, depending on the secondary antibodies used, 2% wt:vol bovine serum albumin, and 0.1% vol:vol TritonX-100 in PBS) for 45 Min at RT. Primary and secondary antibodies (see Antibody table) were diluted in 50% blocking solution in PBS containing either normal goat or donkey serum (depending on the secondary antibodies). Primary antibodies were applied over night at 4°C, followed by three washes in PBS at RT. Secondary antibodies together with DAPI (1:180 dilution of 0.1 mg/mL stock solution, Sigma Aldrich) were applied for 2 h at RT. Fixed whole-mount retinas were incubated over night at 4°C in blocking solution, primary antibodies were applied for 6 days at 4°C and secondary antibodies together with DAPI for 5 days at 4°C. Whole-mount retinas and sections were mounted on slides with DAKO Fluorescence Mounting Medium (Agilent Technologies).

**Table:** 

**Name**	**Host/Class**	**Company**	**Catalog No**.	**Dilution**
Anti GFP	Rabbit/Polyclonal	Invitrogen	A11122	1:500
Anti GFP	Chicken/Polyclonal	Abcam	ab13970	1:500
Anti PKCα	Mouse/Monoclonal	Santa Cruz Biotechnology	sc8393	1:750
Anti Goα	Mouse/Monoclonal	EMD Millipore	MAB3073	1:750
Anti Gγ13	Rabbit/Polyclonal	Santa Cruz Biotechnology	sc368324	1:500
anti GLYT1	Rabbit/Polyclonal	antibodies-online	ABIN1841935	1:500
Anti-Rabbit Alexa Fluor 488	Goat/Polyclonal	Invitrogen	A11008	1:400
Anti-Mouse Cyanine 3	Goat/Polyclonal	Invitrogen	A10521	1:400
Anti-Rabbit Alexa Fluor 488	Donkey/Polyclonal	Invitrogen	A21206	1:400
Anti-Chicken Cyanine 5	Donkey/Polyclonal	Jackson Immuno-Research Laboratories	703-175-155	1:400
Anti-Rabbit Alexa Fluor 594	Goat/Polyclonal	Invitrogen	A11037	1:400

### Microscopy and image analysis

A ZEISS LSM 880 with Airyscan and ZEN 2.1 software was used to take confocal images using either a 20x or 40x objective lens. Images were imported to Fiji (Schindelin et al., 2012) for image processing and cell counting.

### Statistics

Microsoft Excel was used for all statistical calculations. We performed two-tailed Student's *t*-tests to determine significant differences between groups. Data were expressed as the mean ± standard deviation (SD). Different levels of significance are indicated by ^*^ for *P* < 0.05, ^**^ for *P* < 0.01, and ^***^ for *P* < 0.001.

## Author contributions

Conceived and designed the experiments: SK; performed the experiments: MvW, EH, LG, AE; analyzed the data: SK, MvW, EH; contributed reagents/materials/analysis tools: SK, AE; wrote the paper: SK.

## Funding

This work was financially supported by grants from the Haag-Streit Holding AG, the Swiss National Science foundation (31003A_152807/1), the Dr. Streuli-Fonds of the Department of Ophthalmology and a CTU Research Award (84800858) from the Bern University Hospital (Inselspital).

### Conflict of interest statement

The Department of Ophthalmology receives research support from Heidelberg Engineering, and some members of the team are consultants to Heidelberg Engineering.

## Supplementary material

Figure S1 **AAVBP2-treated wild-type mouse retinas show sporadic labeling of B-Type horizontal cells. (A–D)** Examples of B-Type horizontal cells labeled in AAVBP2-treated retinas. In order to demonstrate the classic B-Type horizontal cell morphology, micrographs were taken from areas with low overall levels of transfection where background fluorescence was minimal. Axons are indicated by arrows.

Figure S2 **(A)** Regions of the human retina used for explant culturing. Explants of ~0.5 cm^2^ were prepared from the mid-periphery (indicated in red) or the macula (indicated in blue). **(B,C)** Whole-mount images of explants treated with AAV7m8 **(B)** and AAVBP2 **(C)** stained for mCitrine (green), PKCα (red) and DAPI (blue). Both explants were from the mid-periphery, indicated by the red band in **(A)**.
