# Supplementary figures and images for: Present Molecular Limitations of ON-Bipolar Cell Targeted Gene Therapy
Source: Front Neurosci. 2017 Mar 29;11:161. doi: 10.3389/fnins.2017.00161 (PMC5372788; doi:10.3389/fnins.2017.00161)

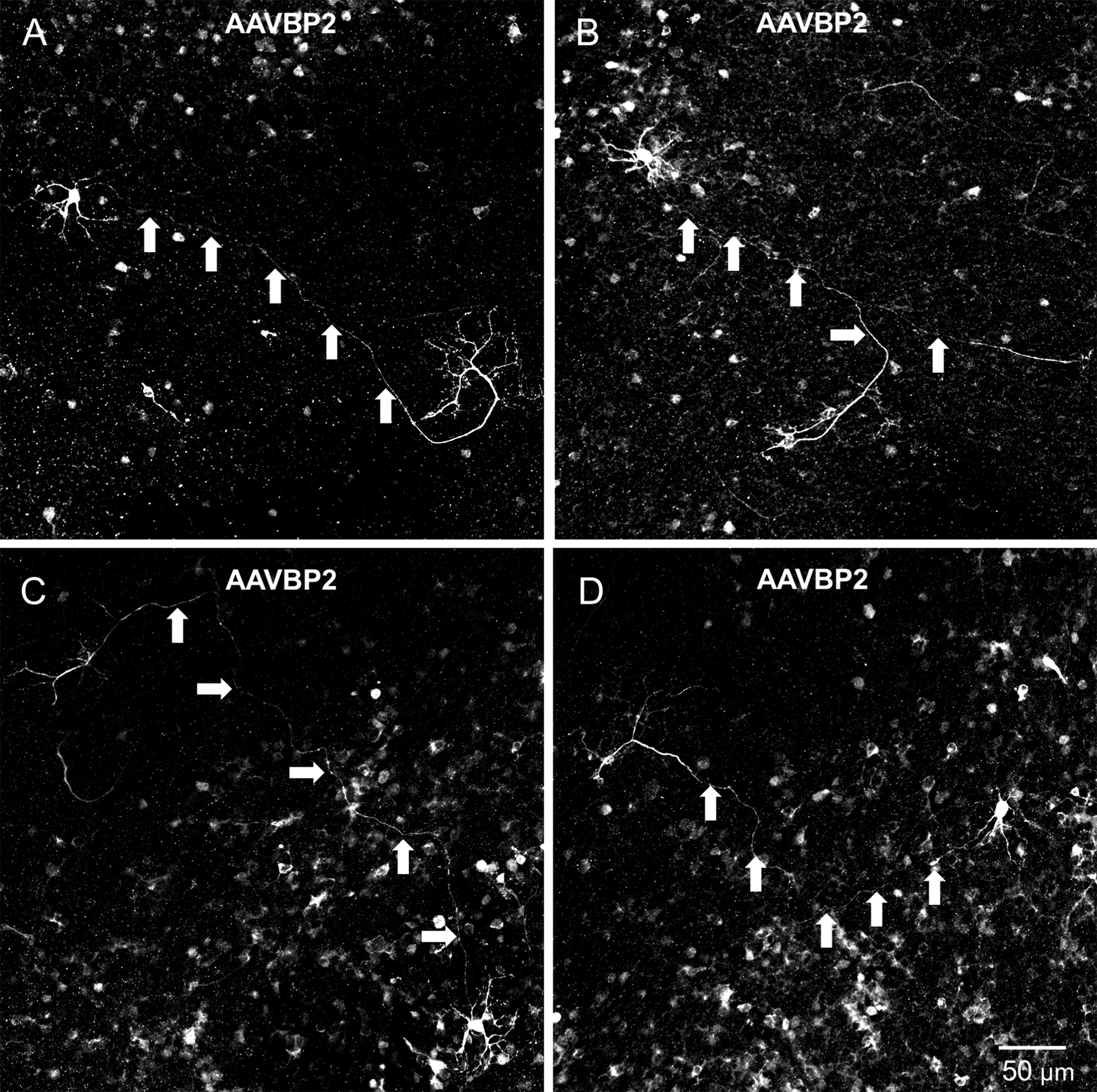

Supplement: Figure S1 — AAVBP2-treated wild-type mouse retinas show sporadic labeling of B-Type horizontal cells. (A–D) Examples of B-Type horizontal cells labeled in AAVBP2-treated retinas. In order to demonstrate the classic B-Type horizontal cell morphology, micrographs were taken from areas with low overall levels of transfection where background fluorescence was minimal. Axons are indicated by arrows. [file Image1.TIFF]

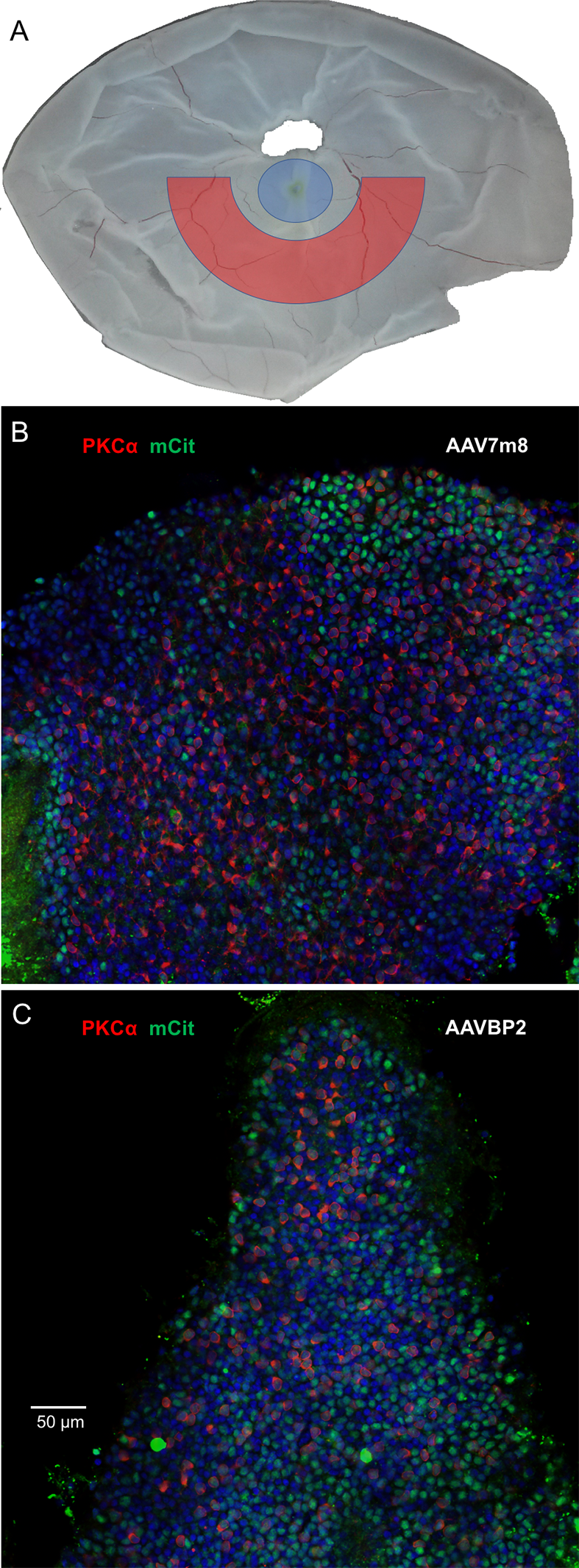

Supplement: Figure S2 — (A) Regions of the human retina used for explant culturing. Explants of ~0.5 cm2 were prepared from the mid-periphery (indicated in red) or the macula (indicated in blue). (B,C) Whole-mount images of explants treated with AAV7m8 (B) and AAVBP2 (C) stained for mCitrine (green), PKCα (red) and DAPI (blue). Both explants were from the mid-periphery, indicated by the red band in (A). [file Image2.TIFF]
